# Two weeks of twice-daily prism adaptation treatment does not improve posture or gait in Parkinson’s disease: a double-blind randomized controlled trial

**DOI:** 10.1186/s13063-021-05832-2

**Published:** 2021-11-25

**Authors:** Janet H. Bultitude, Dawna M. Pidgeon, Pauline R. LeBlanc, Charlotte A. Jeffreys, Faith P. Alexandre, Stephen L. Lee

**Affiliations:** 1 Department of Psychology, University of Bath, North East Somerset, Bath, UK; 2 Department of Neurology, Dartmouth Hitchcock Medical Center, Lebanon, NH USA; 3 Geisel School of Medicine at Dartmouth, Hanover, NH USA

**Keywords:** Parkinson’s disease, Prism adaptation, Gait, Rehabilitation, Clinical trial, Freezing of gait

## Abstract

**Background:**

Gait difficulties in Parkinson’s disease have been related to problems shifting the center of gravity forward. We previously showed reduced forward stepping latencies for people with Parkinson’s disease after one session of adaptation to upward visual shifts, which produces downward motor after-effects and potentially shifts the center of gravity forward. Here we tested if repeated prism adaptation improved gait and postural control in Parkinson’s disease through a parallel, double-blind, randomized, sham-controlled trial.

**Methods:**

We recruited participants with idiopathic Parkinson’s disease aged 40–85 and meeting any one of three clinical criteria: (1) Hoehn and Yahr Stage II.5–IV; (2) scoring > 0 on the gait, freezing of gait, and/or postural stability items of the Movement Disorder Society Unified Parkinson’s Disease Rating Scale; or (3) Timed Up and Go > 12 s. Sealed envelope style randomization allocated participants to two weeks of twice-daily prism adaptation or sham treatment. Participants, care givers, and those assessing the outcomes were blinded to group assignment. Primary outcomes were changes in postural control measured using the Berg Balance Scale and the Limits of Stability, Sensory Organization, and Motor Control tests from the Smart EquiTest system. Secondary outcomes included other physiotherapy and questionnaire measures. Outcomes were assessed at the Dartmouth Hitchcock Medical Center immediately before and after the treatment period, with further long-term postal follow-up over 3 months. Outcomes were analyzed using analyses of variance with follow-up *t* tests.

**Results:**

Eighteen participants were allocated to undergo prism adaptation, of which sixteen were analyzed. Thirteen participants were allocated to undergo sham treatment, and all were analyzed. The prism adaptation group showed increased forward stepping velocity on the Limits of Stability test (pre: *M*=2.33, SEM=0.24; post: *M*=2.88, SEM=0.26; *t*(15)=3.2, *p*=.005, *d*=.819). The sham group showed no such change (pre: *M*=2.13, SEM=0.22; 1d post: *M*=2.24, SEM=0.22; *t*(13)=.636, *p*=.537, *d*=.176). However, there were no group differences for any other outcome measures and no indications that prism adaptation produced functional improvements in posture, gait, or activities of daily living.

**Conclusions:**

Prism adaptation does not improve gait or postural control in Parkinson’s disease.

**Trial registration:**

ClinicalTrials.govNCT02380859. Registered prospectively on 5 March 2015.

**Supplementary Information:**

The online version contains supplementary material available at 10.1186/s13063-021-05832-2.

## Background

Reduced confidence in walking contributes to loss of independence in people with Parkinson’s disease. For example, people with Parkinson’s disease report they have difficulty initiating their gait, and that they “freeze” upon reaching a visual barrier (e.g., doorway [1];). These difficulties have been characterized as problems in shifting the center of gravity (COG) of the body forward and downward over the feet [2, 3]. In neurologically healthy people, walking from standing begins with a forward shift in the COG until it reaches an apex and the person must either take a step forward or fall over [4]. This COG shift is both smaller in people with Parkinson’s disease and related to symptom severity [2]. Compromised ability to shift the body forward therefore appears to be one biomechanical mechanism of impaired initiation and continuation of walking in Parkinson’s disease. Indeed, Martin and Hurwitz [4] observed that some people with Parkinson’s disease could walk more easily when their COG was shifted forward (e.g., by having them carry a chair).

Postural imbalance can also follow unilateral brain lesions. People with brain lesion—particularly to the right hemisphere—can lean towards the ipsilesional side (an ipsilesional shift in COG [5];). Sensory-motor adaptation to a rightward shift in the visual image reduces postural imbalance in people with right hemisphere lesions, restoring central standing posture [6, 7]. In this method—called prism adaptation—patients reach to targets viewed through prismatic lenses that shift the visual image rightward. Patients initially point to the right of the target because their movements are programmed based on the shifted visual information. With repeated movements, however, pointing becomes accurate again, as they are adjusted in the opposite direction of the visual distortion (i.e., to the left). Once the prisms are removed, this realignment is observed as leftward pointing errors (the “adaptation aftereffect” [8];). In people with postural imbalance following right hemisphere lesions, adaptation to rightward-shifting prisms results in a leftward correction of their standing posture [6, 7].

In a previous study, we examined whether gait disturbance in people with Parkinson’s disease could be relieved by adaptation to upward-shifting prisms [9]. Extrapolating from the studies of stroke patients [6, 7], we reasoned that adaptation to upward-shifting prisms would induce a forward and downward postural shift that would assist people with Parkinson’s disease to attain greater forward propulsion. In our study [9], we recorded the forward and backward stepping initiation time of neurologically healthy participants and people with idiopathic Parkinson’s disease before and after a single session of prism adaptation. We found that the time taken to initiate forward steps was significantly reduced after prism adaptation, for both people with Parkinson’s disease and age-matched controls. Backward stepping times were unchanged. These results therefore provided evidence that adaptation to upward-shifting prisms could selectively reduce forward stepping time. Research involving stroke patients suggests that repeated daily sessions of prism adaptation are required to impart sustained, clinically significant improvements [10–13, 31]. Therefore, building on our proof-of-principle evidence [9], the objective of this study was to test the effect of a two-week regimen of twice-daily prism adaptation on posture and gait in people with Parkinson’s disease in a double-blind, randomized, sham-controlled trial.

To determine whether prism adaptation would result in functional benefits, we assessed posture, gait, and activities of daily living in one session before and one session after the treatment period using self-report questionnaires, standard clinical assessments, and mechanized measurements. To evaluate any long-term effects of prism adaptation, we administered further follow-up questionnaires 1 week, 1 month, and 3 months post-treatment.

We hypothesized that, compared to patients who undergo sham prism adaptation, patients who completed 2 weeks of real prism adaptation would show reduced stepping initiation times, greater displacement of the body over the feet during stepping initiation, and increased self-reported confidence while walking. We also measured a number of other clinical and self-report indicators to determine whether prism adaptation improved motor performance and postural responses to external perturbations.

## Methods

### Design

The study had a double-blind randomized controlled design with two parallel groups. The intended allocation ratio was 1:1 (real:sham); however, the final allocation ratio was 1.38:1 due to a deviation from the intended allocation procedure (see the “Randomization and allocation” section).

A summary of the study sessions and procedures is provided in Table 1. The participants attended two research visits lasting 3–4 h at the Dartmouth-Hitchcock Medical Center (the “pre” test and “one day post” test; 1d post). In the 14 days between these sessions (the “treatment period”), participants completed twice daily prism/sham adaptation at home. In the first research visit, the participants underwent a neurological and cognitive assessment to confirm eligibility for the study, and provided informed written and verbal consent. In both research visits, participants completed self-report questionnaires, and their posture and gait were assessed using standard clinical tests and mechanized measurements. Symptoms were also assessed 1 week (“one week post”; 1w post), 1 month (“one month post”; 1m post), and 3 months (“three months post”; 3m post) after the end of treatment using the self-report questionnaires, sent by post. The participants, and the researchers responsible for participant enrolment and assessing outcomes (PRLeB, CAJ, DP, and SLL) were all blinded as to which type of treatment each participant underwent. The trial ended when all enrolled participants had either returned the final self-report questionnaires, withdrew, or were deemed lost to follow-up.

**Table 1:** Timing of study sessions and procedures

Session	Pre (in person)	Treatment (at home)	1d post (in person) Primary endpoint	1w post (mail)	1m post (mail)	3m post (mail)
**Timing**	Day 1	Days 2–14	Day 15	Week 3	Week 7	Week 15
**Activity**						
Informed consent	x					
Demographics	x					
*Neurologist assessment*						
General Neurological Exam	x					
Montreal Cognitive Assessment	x					
Unified Parkinson’s Disease Rating Scale	x					
*Physical therapist assessment*						
Time Up and Go Test	x		x			
Functional Gait Assessment	x		x			
Berg Balance Scale	x		x			
Mechanized measurements (SMART EquiTest Balance Master®)	x		x			
*Questionnaires*						
Fall Efficacy Scale	x		x	x	x	x
Activities-specific Balance Confidence Scale	x		x	x	x	x
Parkinson’s Disease Questionnaire-39	x		x	x	x	x
New Freezing of Gait Questionnaire	x		x	x	x	x
Patient-Centered Outcome Questionnaire-Parkinson’s disease	x					
Patient’s Global Impression of Change			x	x	x	x
*Treatment-related procedures*						
Randomization and allocation	x					
Prism adaptation training	x					
Real/sham adaptation treatment (2x per day)		x				
Logbook		x				
Post-treatment interview (guess condition)			x			

### Participants

**Fig. 1:**
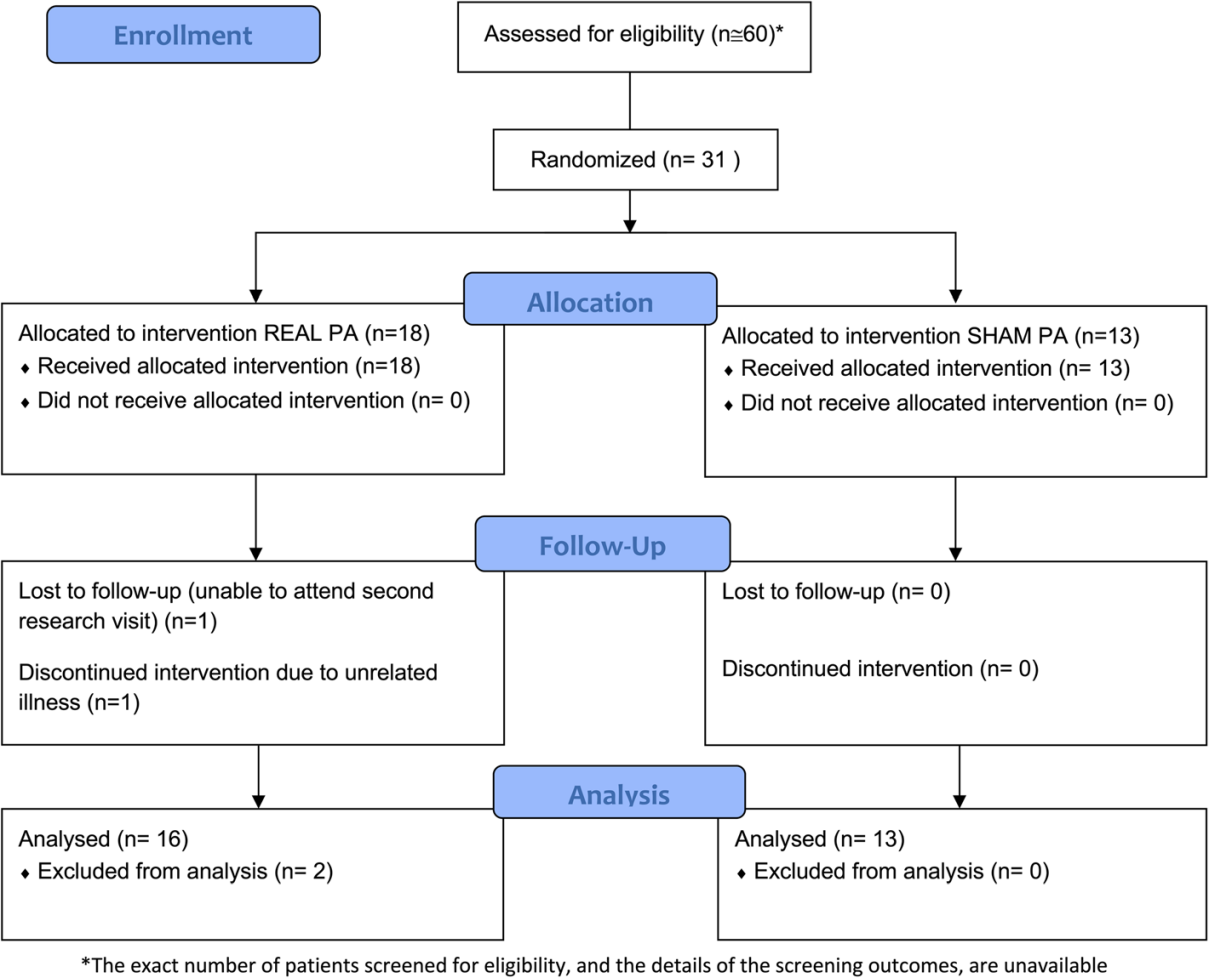
Participant flow through the study

Participants with idiopathic Parkinson’s disease were recruited between April 2015 and February 2017 from among patients referred to the Balance and Vestibular Centre of the Dartmouth-Hitchcock Medical Center for gait training (Fig. 1). The target sample size was 32, based on pragmatic considerations of the number that could be recruited and tested given the available time and resources, and sample sizes for two-branch studies about prism adaptation treatment in stroke patients [11–13]. We recruited 31 patients into the study in the time available. Inclusion criteria were being aged 40–85 inclusive and meeting any one of three clinical criteria: (1) Hoehn and Yahr Stage II.5–IV; (2) scored > 0 on any of the gait, freezing of gait, and postural stability items of the Movement Disorder Society Unified Parkinson’s Disease Rating Scale [14] (MDS-UPDRS Items III.10–III.12); or (3) Timed Up and Go test duration > 12 s. Exclusion criteria were (1) known psychiatric or neurological condition other than Parkinson’s disease that in the opinion of the assessing Neurologist (SLL) would compromise participation in the study; (2) known neurological diagnosis, other than Parkinson’s disease, that can cause imbalance and gait impairment (e.g., multiple sclerosis, stroke); (3) normal score on the MDS-UPDRS part III; (4) injury or impairment to the right arm that would substantially affect pointing movements (other than that due to Parkinson’s disease); (5) lacking sufficient visual acuity to view the target and hand during prism adaptation; and (6) lacking sufficient understanding of verbal and written English to give informed consent.

Participants who were taking CNS-acting medications (e.g., benzodiazepines, hypnotics, antidepressants) were on a stable regimen for at least thirty days. One participant withdrew because of illness unconnected to the intervention, and another because they were unable to attend visit 2.

A neurologist with over 15 years’ experience in assessing and managing Parkinson’s disease (SLL) conducted a neurological assessment at the pre-test. The assessment included (1) a standard neurological examination, (2) the Montreal Cognitive Assessment (MoCA) [15], and (3) evaluation of Parkinson’s disease severity using the motor component of the MDS-UPDRS (Part III) [14]. Participants also completed the Patient-Centered Outcomes Questionnaire for Parkinson’s Disease [16] at baseline so that the perspectives and expectations about possible treatment success could be compared between groups, since these can impact on trial outcomes.

### Outcomes

The effect of prism adaptation on postural control (primary outcomes) and gait (secondary outcomes) was measured by a physical therapist with over 20 years’ experience in vestibular rehabilitation and falls prevention (DMP), including daily use of the described outcome measures as part of her standard clinical practice.

#### Primary outcome measures: change in postural control

##### Berg Balance Scale [17]

This 14-item test assesses static and dynamic balance. The assessor rates performance on each item from 0 (“lowest level of function”) to 4 (“highest level of function”) and calculates a total out of 56.

#### Mechanized measures of postural control

The Smart Equitest Balance Master System® (NeuroCom Int., Inc., Clackamas, Oregon) consists of two parallel force plates that detect the center of pressure through four force transducers connected to a computer system. The force plates are enclosed on the front, left, and right by a visual surround. Visual instructions and feedback are provided via a computer monitor embedded in the visual surround. The force plate can move laterally and in the anterior/posterior plane, and both the force plate and the visual surround can rotate in the sagittal plane.

The following tests were administered using the procedures outlined in the SMART EquiTest system manual version 8:

##### Limits of stability test

This measures a person’s ability to displace COG relative to their feet without losing balance or taking a step. The participant stood on the platform in front of a monitor positioned approximately 58 cm away at eye level. Nine squares on the monitor represented locations relative to the upright starting position: one in the center of the screen (upright) and the remaining eight forming an ellipse around the central square representing locations forward, backward, and to the left and right of the participant as well as all four diagonals. The participant’s COG was represented on the screen by an avatar. At the beginning of each trial, the participant was instructed to stand upright, keeping the COG cursor over the central square. After 2000 ms a circular target appeared in one of the other squares. The participant was required to move the COG cursor towards the second target as quickly and accurately as possible by leaning their body without moving their feet and then holding a position as close to the target as possible. The participant completed one trial per location in clockwise order. Up to 8 s was allowed for each trial. Reaction time, maximum movement velocity, and maximum extension of the COG over the feet were recorded. Only stepping in the forward and backward direction were considered relevant for the study’s hypotheses.

##### Sensory Organization Test

This measures postural control under different somatosensory, visual, and vestibular feedback conditions. The participant is asked to stand quietly and maintain an upright posture. Proprioceptive feedback to the feet and joints is manipulated by allowing the platform to tilt to directly follow the participant’s anteroposterior body sway (constant proprioception) or by maintaining the standing platform in a fixed horizontal position (normal proprioceptive feedback). Visual feedback is varied by asking the participant to close their eyes (no visual feedback), asking the participant to open their eyes and allowing the visual surround to tilt directly following the participant’s anterioposterior body sway (constant visual feedback), or by asking the participant to keep their eyes open and maintaining the visual surround in a fixed position (normal visual feedback). A composite equilibrium score is provided that quantifies the overall COG sway or postural stability across six feedback conditions. In addition, COG alignment in the left-right and forward-backward planes are provided, reflecting the resting COG relative to the base of support at the beginning of each trial. Only the COG alignment in the forward-backward plane was considered relevant for the study’s hypotheses.

##### Motor Control Test

This assesses the ability of the automatic motor system to recover following an unexpected external disturbance. Sequences of small, medium, or large platform translations in forward and backward directions elicit automatic postural responses. Translation of the surface in one horizontal direction results in displacement of the COG away from the center in the opposite direction relative to the base of support. To restore a normal balance, a quick movement of the COG back to the center position is required. A composite latency value is provided as the time in milliseconds between the force plate translation and the patient’s corrective responses, averaged across all translation sizes and directions.

#### Secondary outcome measures: changes in gait and self-reported questionnaires

##### Timed Up and Go test

Patients are seated in a chair and instructed to stand up, walk 3 m, turn around, walk back, and sit down. The time (in seconds) is recorded.

##### Functional Gait Assessment [18, 19]

This 10-item test assesses dynamic balance and postural stability during gait. The assessor scores the patient’s performance from 0 (“severe impairment”) to 3 (“normal”) for each item and calculates a total out of 30.

##### Walk Across

This quantifies characteristics of steady state gait as the patient walks across the length of the force plate of the Smart Equitest Balance Master System. Average step length and speed were recorded.

Four questionnaires were administered at the pre-test and each of the four post-tests (see Table 1). An adapted version of the Patient’s Global Impression of Change Questionnaire was also administered at the four post-tests.

##### New Freezing of Gait Questionnaire [20]

This nine-item questionnaire evaluates the impact and severity of freezing of gait on locomotion and daily activities. Items are scored from 0 or 1 to 3 or 4, with higher numbers indicating greater impact and severity. A total is calculated out of 28.

##### Falls Efficacy Scale [21]

This ten-item questionnaire asks people to rate their confidence in performing daily activities without falling. Responses are given on a scale from 0 (“very confident”) to 10 (“not very confident”) and a total is calculated out of 100.

##### Activities-specific Balance Confidence Scale [22]

This 16-item questionnaire is similar to the Falls Efficacy Scale, but has greater sensitivity to gait impairments in more ambulatory patients. Participants rate their confidence that they will not lose their balance or become unsteady when performing specific activities. Responses are given on a scale from 0 to 100% in 10% increments where 0% is “no confidence” and 100% is “completely confident.” An average is calculated.

##### Parkinson’s Disease Questionnaire 39 [23]

This 39-item questionnaire is the most widely-used Parkinson’s disease-specific measure of health and daily function. Participants rate the extent to which they experience problems with aspects of mobility and self-care from 0 (“never”) to 4 (“always”). A percentage is calculated. In addition to the overall percentage, the mobility (questions 1–10) and activities of daily living (questions 11–16) sub-sections were analyzed separately to assess changes in these specific outcomes.

##### Patient’s Global Impressions of Change [24]

This measures the patient’s self-reported change in symptoms. Patients are asked to separately rate “the change (if any) in ACTIVITY LIMITATIONS, SYMPTOMS, EMOTIONS, and OVERALL QUALITY OF LIFE” related to their condition on a 7-point Likert scale from 1 (“no change”) to 7 (“a great deal better”). In addition to the standard question, we also asked patients to give three additional ratings indicating their perceived change in freezing of gait, walking, and balance.

### Intervention

The intervention involved making pointing movements to visual targets while wearing goggles fitted with Fresnel lenses that induced a 25-dioptre (~ 17°) upward shift in the visual image (“real” treatment), or that induced no shift (“sham” treatment). At the end of the first research session, a researcher who was not involved in assessing baseline symptoms or outcomes (JHB or FPA) trained the participant in the treatment and guided them through one treatment session. Regardless of treatment, the researcher stated that the goggles distorted vision, “for example, they restrict your field of vision.” Participants were seated in a chair within arms’ reach of a wall while wearing the goggles. A sheet of US letter-sized paper (the “target sheet”) was secured to the wall in portrait orientation, upon which were two 2-cm-diameter targets spaced 25 cm apart, one above the other. Participants were instructed to use the index finger of their right hand to touch each target alternately, bringing their finger to their chest between each pointing movement. They were instructed to perform the pointing movements as fast as possible with minimal head movements for 50 total movements (25 per target), and then to remove the goggles.

After the participant completed the treatment and the researcher was satisfied that they understood the instructions, the participant was provided with the target sheet, written instructions, a weblink to a video demonstration of the treatment session (as performed by an actor), contact information for the researcher, and a logbook. Participants were instructed to perform the treatment at home twice a day (morning and evening), starting with the morning of the following day. Participants could perform the treatment alone or with a caregiver, according to their preference. They were asked to record in the logbook the time and duration of each treatment, and their perceived levels of attention, fatigue, and effort during each treatment. Participants were asked not to alter any other treatments or rehabilitation techniques that they were engaged with during the course of the study period, however aside from medications (Supplementary Tables 1 and 2), we did not record any co-interventions participants were using (e.g. physiotherapy and strength training). Aside from the logbook entries, there were no other efforts to monitor adherence during the intervention period, although we emphasized that the participants could contact the researchers at any time with questions.

### Randomization and allocation

The participants were allocated to a treatment group during the first research visit by the researcher who trained them in performing the treatment. The participants were randomized to undergo real or sham prism adaptation through a sealed envelope-style blind draw. At the beginning of the study, sixteen of each type of goggles were placed by JHB in individual opaque zip-lock bags and mixed together in the same box. To allocate each patient, the researcher responsible for treatment training (JHB or FPA) extracted a set of goggles from the box. It was intended that this allocation procedure would be used until each set of goggles had been provided to a participant, resulting in two groups of 16 patients. However, some goggles were returned to the box after previous participants were finished with them. That is, instead of the goggle allocation occurring without replacement of previously used goggles into the box, the allocation occurred *with* replacement. This resulted in 18 patients undergoing real adaptation and 13 undergoing sham adaptation. Of these, two patients allocated to the real adaptation condition withdrew from the study (Fig. 1).

## Results

The full research data are provided in Additional file 1.

### Group matching, compliance, and success of participant blinding

The groups had comparable baseline patient characteristics (Table 2; individual patient characteristics provided in Supplementary Tables 1 and 2 in Additional file 2). There were no baseline differences on the New Freezing of Gait questionnaire; Falls Efficacy Scale; Activities-specific Balance Confidence Scale; mobility, Activities of Daily Living, or total scores on the Parkinson’s Disease Questionnaire 39; or the Patient-Centered Outcomes Questionnaire for Parkinson’s Disease (*p*s >.094). The groups did not differ in the number of training sessions self-reported as completed according to their log books [sham group: *M*=27.0, SEM=0.40; real group: *M*=26.4, SEM=0.68; *t*(27)=0.7, *p*=.462)]. The groups also did not differ in self-rated attention, fatigue, and effort during treatment (*p*s ≥ 0.217). There was no difference in the percentage of people in each group who chose “sham,” “real,” or “no idea” when asked to guess the treatment that they had been assigned to in the post-treatment session [sham group: 54%/31%/15%; real group: 37.5%/37.5%/25%; *Χ*^2^ (2) = 3.8, *p*=.149]. For those patients who guessed sham or real, the confidence (%) of their guesses were not different between the groups (guessed sham: *M*=68.8, SEM=4.5; guessed real: *M*=78.5, SEM=6.3; *t*(19)= 1.3 *p*=.216). No participants reported any harms or unintended effects.

**Table 2:** Baseline demographic and clinical characteristics, split by group

	Real^**a**^	Sham^**a**^	Comparison
N	16	13	
Sex (% females)	31.3	53.8	^b^*p*=.19
Age (years)	70.8 (7.9)	68.3 (8.2)	*t*(27)=0.83, *p*=.41
Handedness (% left)	0.0	23.1	*Χ*^*2*^(29)=4.1, *p*=.042^c^
Time since diagnosis (years)	7.5 (5.8)	7.7 (5.3)	*t*(27)=0.14, *p*=.89
Weight (pounds)	171.0 (58.5)	159.9 (38.2)	*t*(27)=0.59, *p*=.56
Height (inches)	66.6 (4.4)	66.5 (5.1)	*t*(27)=0.01, *p*=.99
MOCA (/30)	24.7 (0.5)	26.5 (3.0)	*t*(27)=1.5, *p*=.15
*MDS-UPDRS-III*			
Gait (/4)	1 (1)	1 (2)	*U*(29)= 84.5, *p*=.31
Freezing (/4)	0 (1)	1 (0.5)	*U*(29)= 87.5, *p*=.48
Postural stability (/4)	1 (0.75)	1.0 (1.0)	*U*(29)= 83, *p*=.37
Total (/132)	34.3 (9.9)	33.5 (14.7)	*t*(27)=0.12, *p*=.87
Hoehn and Yahn	2.25 (1)	2.5 (1)	*U*(29)= 89.5, *p*=.53
TUAG (s)	11.6 (5.0)	9.9 (3.0)	*t*(27)=0.93, *p*=.26

Abbreviations: *MDS-UPDRS-III*, Movement Disorder Society Unified Parkinson’s Disease Rating Scale section three; *MOCA*, Montreal Cognitive Assessment; *TUAG*, Timed Up and Go. ^a^Summary statistics indicate percentage, mean (standard deviation), or median (interquartile range). ^b^Fisher’s exact test. ^c^Significant

Questionnaire return rate was higher than 87% across all five testing points, and higher than 97% for the pre and 1d post test. Of the questionnaires that were returned, missing data due to incomplete items were negligible (9 data points across five questionnaires). Missing data were replaced with the group mean for that item. One patient (VA32) arrived late for the 1d post test, leaving insufficient time to run assessments for the Berg Balance Scale, Sensory Organization Test, and Motor Control Test. Since partial post-treatment data were available for this patient, their missing values were replaced with the group mean. Additional missing data constituted less than 5% of the data and were replaced with group means.

### Overview of treatment outcomes

**Fig. 2:**
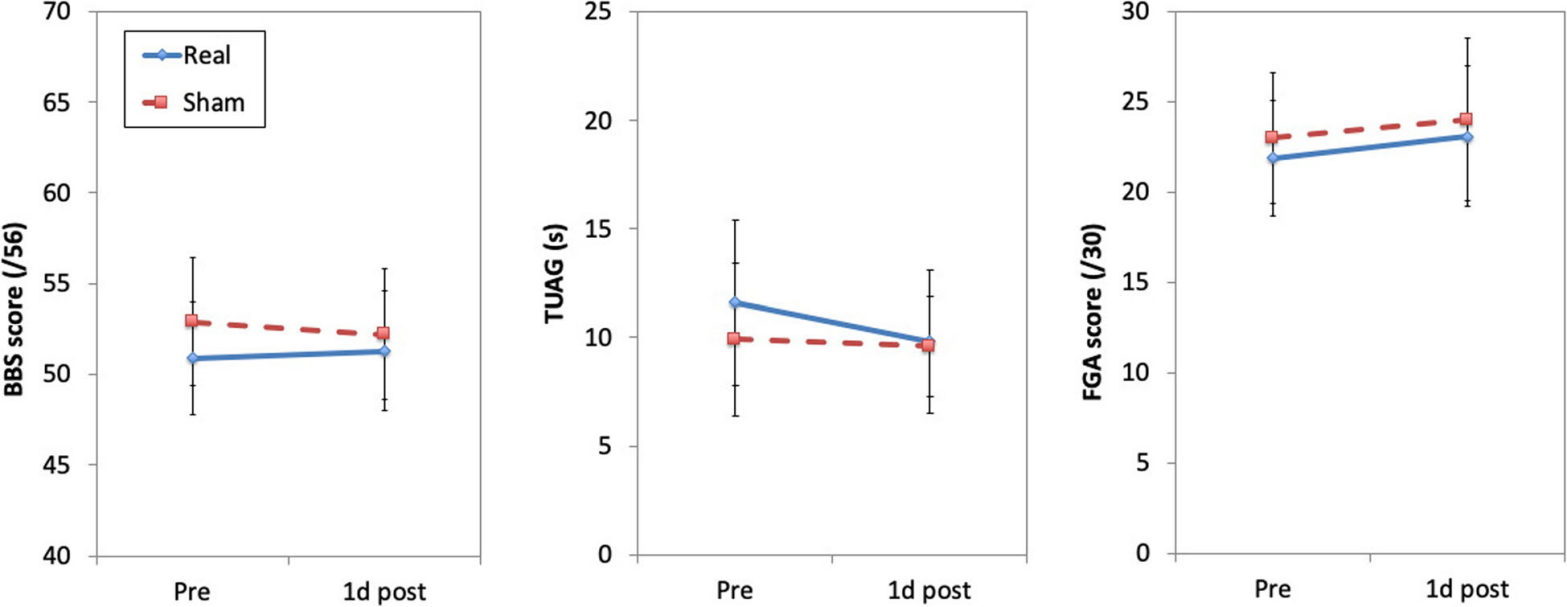
Results for the Berg Balance Scale, Timed Up and Go, and Functional Gait Assessment. Abbreviations: BBS, Berg Balance Scale; TUAG, Timed Up and Go; FGA, Functional Gait Assessment. Error bars represent ±1 SEM

**Fig. 3:**
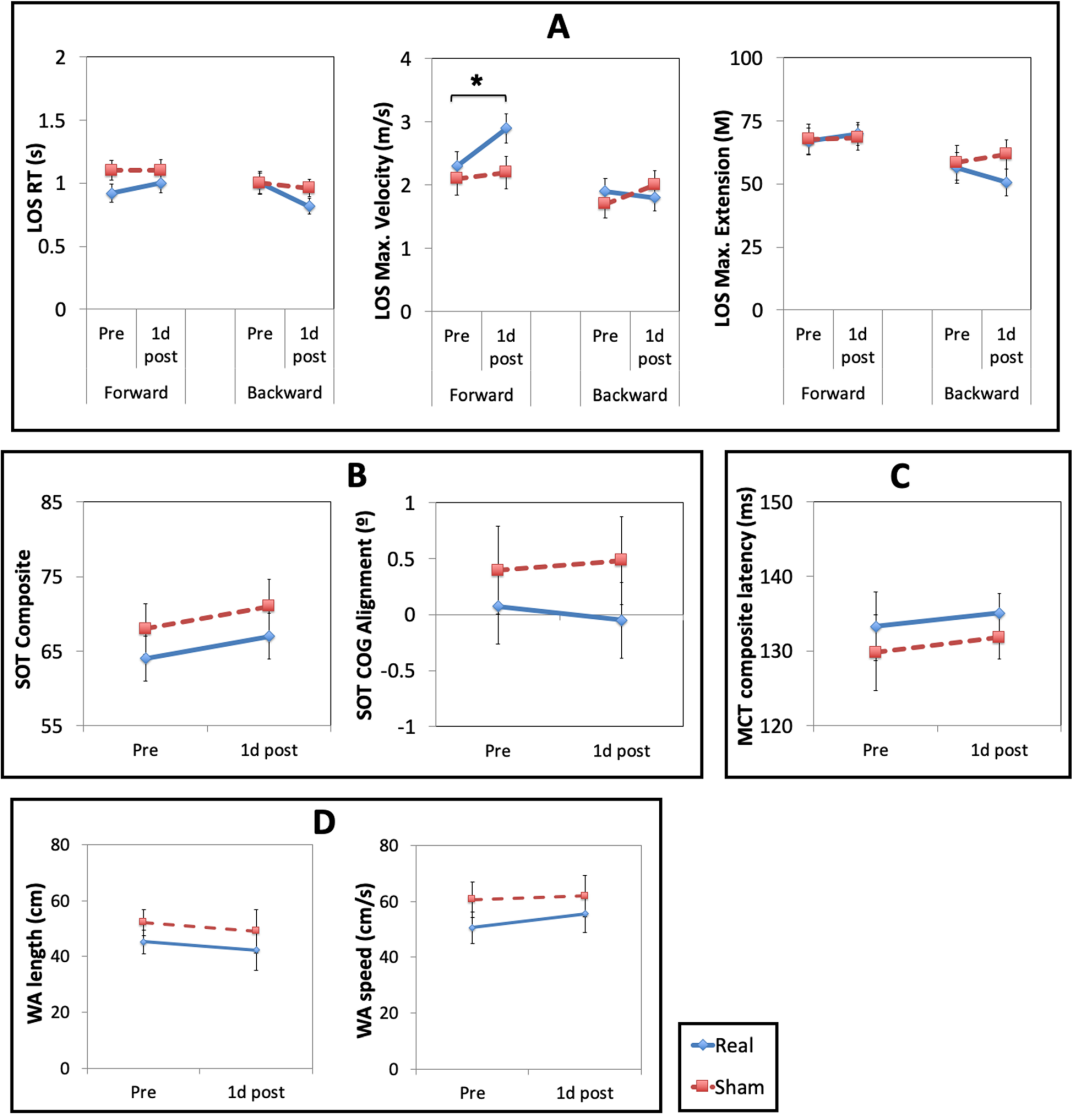
Results of mechanized measures for the real and sham treatment groups. **A** Mean reaction time (RT), maximum velocity, and maximum extension on the Limit of Stability (LOS) test for forward and backward stepping, **B** Mean composite scores and forward-backward center of gravity (COG) alignment on the Sensory Organization Test (SOT), **C** Mean composite latency scores on the Motor Control Test (MCT), **D** Mean step length and step speed on the Walk Across (WA) test. * = p < .05. Error bars represent ±1 SEM

Figures 2 and 3 illustrate the results of the mechanized measures, and other physiotherapist assessments, respectively. The results, reported in detail below, revealed almost no differences between the real and sham treatment groups on treatment outcomes. The exception is that the real treatment group had a significant increase in maximum velocity for forward stepping after treatment (based on the Limits of Stability test), whereas the sham treatment group had no such change. Overall, the weight of evidence supported no significant change in posture or gait in either group.

#### Primary outcome measures: change in postural control

##### Berg Balance Scale (Fig. 2)

A group (real, sham) × time (pre, 1d post) ANOVA revealed no main effects or interactions (*F*s ≤1.4, *p*s ≥ 0.25).

##### Mechanized measures

We performed three group (real, sham) × time (pre, 1d post) × stepping direction (forward, backward) ANOVAs of reaction time, maximum velocity, and maximum extension on the Limits of Stability test (Fig. 3A). There was a main effect of direction for maximum velocity (*F*(1,27) = 17.7, *p*<.001, *η*_*p*_^*2*^ = .367; forward: *M*=2.4, SEM=0.16; backward: *M*=1.8, SEM=0.12) and for maximum extension (*F*(1,27) = 15.8, *p*<.001, *η*_*p*_^*2*^ = .369; forward: *M* = 68.2, SEM=3.6; backward: *M*=56.9, SEM=4.1), reflecting faster, and further, forward than backward stepping. There was also a group × time × direction interaction for maximum velocity *(F*(1,27)=5.22, *p*=.03, *η*_*p*_^*2*^ = 1.62). Follow-up *t* tests revealed that the real treatment group showed increased maximum velocity for forward stepping following treatment (pre: *M*=2.33, SEM=0.24; 1d post: *M*=2.88, SEM=0.26; *t*(15)=3.2, *p*=.005, *d*=.819). There were no significant changes in forward stepping for the sham treatment group (pre: *M*=2.13, SEM=0.22; 1d post: *M*=2.24, SEM=0.22; *t*(13)=.636, *p*=.537, *d*=.176) nor in backward stepping for either treatment group (*t*s < 1.68, *p*s >.118). There were no further main effects or interactions in the analyses for the Limits of Stability test (*F*s ≤4.0, *p*s ≥.056).

Group (real, sham) × time (pre, 1d post) ANOVAs revealed no main effects or interaction in the analyses of the composite score (*F*s ≤3.5, *p*s≥.074) and forward-backward displacement of COG (*F*s ≤0.72, *p*s≥.40) from the Sensory Organization Test (Fig. 3B) nor for composite latency in the Motor Control Test (*F*s ≤.61, *p*s≥.441; Fig. 3C).

#### Secondary outcome measures: change in gait and self-reported questionnaires

Group (real, sham) × time (pre, 1d post) ANOVAs revealed no main effects or interactions (*F*s≤2.72, *p*s≥.11) for the Timed Up and Go test, the Functional Gait Assessment (Fig. 2), and mean step length and mean step speed in the Walk Across test (*F*s≤.92, *p*s≥.35; Fig. 3D).

**Fig. 4:**
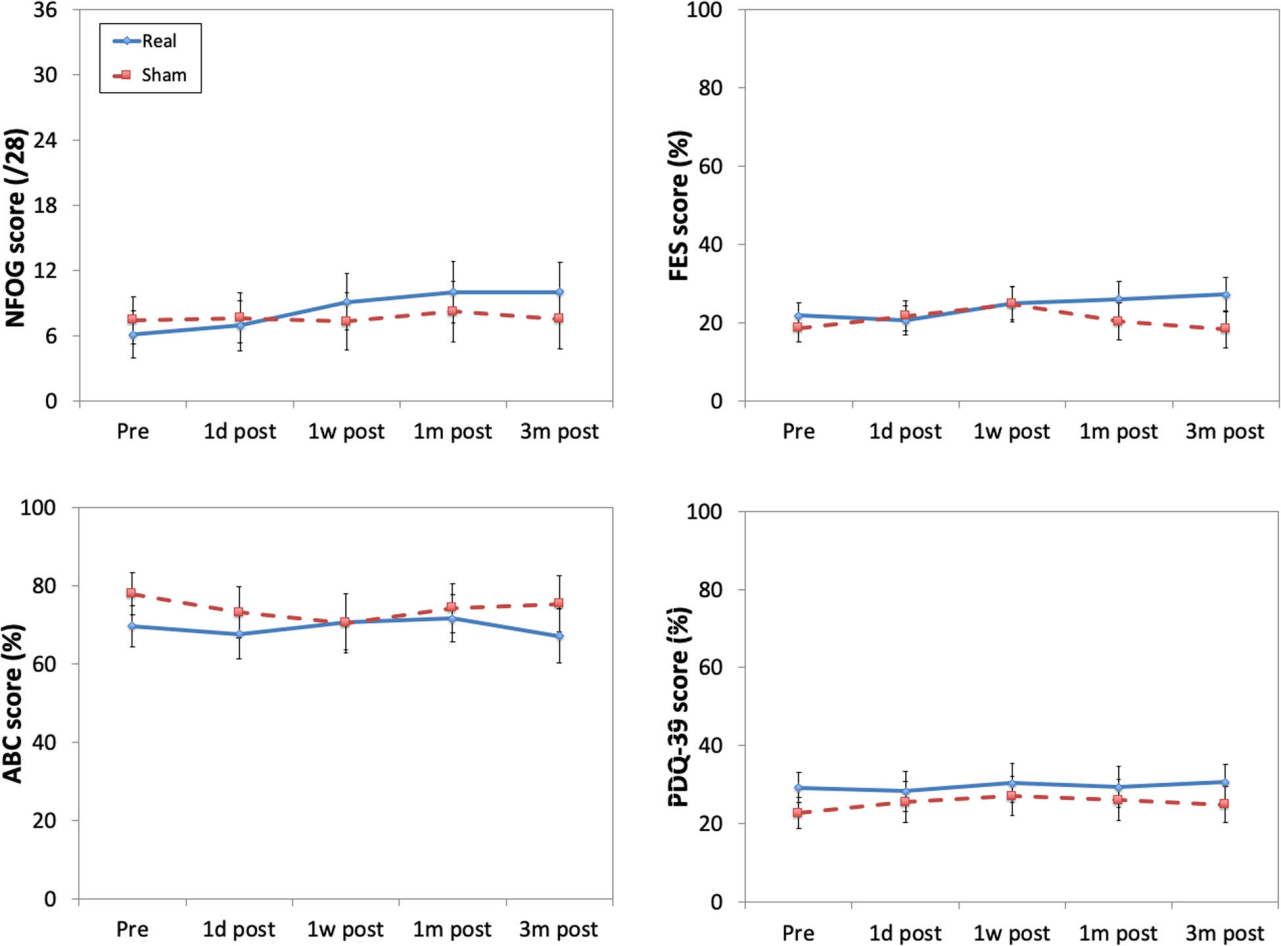
Results for questionnaire measures. Abbreviations: NFOG, New Freezing of Gait Questionnaire; RES, Falls Efficacy Scale; ABC, Activities-specific Balance Confidence scale; PDQ-39, total Parkinson’s Disease Questionnaire 39. Results of the analysis of the mobility and activities of daily living subscales of the PDQ-39 were qualitatively similar to those for the total PDQ-39. Greater symptom severity or impact on activities is indicated by higher scores on the ABC, and lower scores on the other three measures. Error bars represent ±1 SEM

Group (real, sham) × time (pre, 1d post, 1w post, 1m post, 3m post) ANOVAs were performed on the total scores of the New Freezing of Gait Questionnaire; Falls Efficacy Scale; Activities-specific Balance Confidence scale; and the mobility, activities of daily living, and total scores on the Parkinson’s Disease Questionnaire 39 (Fig. 4). There were no main effects or interactions for any analysis (*F*s ≤2.27, *p*s ≥.14)

Patient’s Global Impressions of Change ratings for overall function, walking, freezing, and balance were lower than 3 (“a little better”) across all time points and both groups, indicating little perceived change. A group (real, sham) × time (1d post, 1w post, 1m post, 3m post) × measure (overall, freezing, walking, and balance) ANOVA was performed. There was a main effect of group (*F*(1, 27) = 5.4, *p*=.028, *η*_*p*_^*2*^ = .17): the real treatment group (*M*=1.6, SEM=0.19) perceived less change than the sham treatment group (*M*=2.3, SEM=.22). None of the other main effects or interactions was significant (*F*s ≤2.5, *p*s >.064).

## Discussion

We aimed to examine whether two weeks of twice-daily adaptation to upward-shifting prisms improved posture, gait, and activities of daily living in people with Parkinson’s disease. The results demonstrate that the real treatment group did not improve on clinical or self-report measures compared to the sham treatment group. Thus, prism adaptation does not appear to benefit people with Parkinson’s disease.

The only measure for which people in the real treatment group showed improvement compared to people in the sham treatment group was in forward stepping velocity (Fig. 3A). This resembles our previous finding of shorter forward stepping times following a single session of prism adaptation in people with and without Parkinson’s disease [9]. In the present study, this increase in forward stepping velocity did not translate into any improvements on self-reported or clinically assessed postural control, gait, or function. It is possible that the faster forward stepping speed induced by prism adaptation was not sufficient to improve daily function, or that forward stepping speed does not directly relate to functional measures. It is also possible that selecting patients based on narrower criteria in terms of age and Parkinson’s disease severity might have led to different results (e.g., significant treatment effects might have arisen if only people with mild or severe symptoms were selected). The results of the Sensory Organization Test showed that COG alignment in the forward-backward direction was unchanged by prism adaptation, indicating that the increased velocity of forward stepping occurred without concomitant change in COG at rest. If shifting COG forward is necessary to improve freezing and gait, as was the premise of this study, then two weeks of treatment with adaptation to upward shifting prisms does not appear to be an effective means to achieve this change.

We did not measure adaptation after-effects, thus we must consider the possibility that the lack of benefit of prism adaptation is because people in the real treatment group did not adapt. However, this is unlikely. Although there are documented deficits in sensorimotor adaptation in people with Parkinson’s disease [25–27], these are not sufficient to completely prevent the adaptation that normally arises during a multi-session regimen such as the one we used. In our previous study [9], we recorded significant after-effects after only a single session of adaptation to upward shifting prisms. Also, in the present study, we confirmed during the in-person training that the participants in the present study could complete the treatment as instructed. We are confident that they could continue the treatment at home, given its simplicity; however, an improvement to our approach could have been to have the participants demonstrate how they had performed the treatment when they returned to the hospital for the second in-person research visit. We also note that although we asked participants not to alter their engagement with or use of other rehabilitation methods during the study period, we did not record any co-interventions that participants were using. Although random group allocation means it is likely that the number and type of any co-interventions would have been relatively balanced across groups, we cannot be completely certain that the lack of observed effects of prism adaptation is because of higher engagement of the sham treatment group in other therapies.

Researchers studying prism adaptation for stroke rehabilitation have identified the need to better understand dose optimization [28, 29]. Given that we found a significant increase in forward stepping velocity for the real treatment group, future research could be to test whether this would translate to meaningful changes in postural control, gait, or function over a longer treatment period or with adaptation to larger visual shifts. However, we also speculate that the lack of efficacy of upward-shifting prisms in this study, given the significant effect of rightward-shifting prisms on postural control in stroke patients [6, 7], might be due to prism direction. To the best of our knowledge, no other researchers have examined adaptation to upward visual shifts. There is some evidence that the sensorimotor system has in-built models for adapting to horizontal mirror reversals in vision, but not for vertical reversals—possibly due to the left-right symmetry of the body [30]. This could mean that adaptation to vertical compared to horizontal visual shifts are qualitatively different.

Strengths of the study are that it had a low drop-out rate, successful participant blinding, and good adherence based on the number of logged sessions. Greater confidence in adherence to and correct execution of the treatment could be gained by having a researcher guide the participants through every treatment session; however, we lacked resources for this. We also ruled out asking the patients to teleconference with a researcher, or video record each treatment session, because we considered that such additional complications could be barriers to participation and adherence. We also considered it important to study the effects of a regimen of prism adaptation delivered in the format that would likely be used if it was ultimately used therapeutically. Prism adaptation for gait disturbance in Parkinson’s disease would most likely be an adjunct treatment performed at home in a self-guided manner, similar to physical therapy. We therefore tested prism adaptation in the form in which it would most feasibly be delivered. Nonetheless, future research could make greater efforts to verify that the treatment was performed accurately by the participants by having researchers observe the treatment session as described above, asking caregivers to video record treatment sessions at random intervals for later analysis, or by asking caregivers to complete a simple checklist about the essential features of the treatment at random intervals.

## Conclusions

There were no differences in the effects of prism adaptation compared to sham adaptation on postural control, gait, or function. These results indicate that 2 weeks of twice-daily adaptation to upward-shifting prisms is not an effective treatment for gait disturbance in people with Parkinson’s disease. Prism adaptation did lead to faster forward stepping times whereas sham adaptation did not, therefore there could be merit in investigating whether this translates to functional improvements over a longer treatment period. Overall, however, we found no evidence that prism adaptation should be incorporated into the management of gait disturbance in people with Parkinson’s disease.

## Supplementary Information

**Additional file 1:.** Full study data. Tab S1: Patient Centred Outcome Questionnaire for Parkinson’s Disease. Tab S2: Primary outcomes (Berg Balance Scale, Limits of Stability test, Sensory Organization test, Motor Control test). Tab S3: Secondary outcomes, physiotherapy assessed (Timed Up and Go, Functional Gait Assessment, Walk Across test). Tab S4: Secondary outcomes, questionnaire measures (New Freezing of Gait questionnaire, Falls Efficiency Scale, Activities-specific Balance Confidence Scale, Parkinson’s Disease Questionnaire 39). Tab S5: Secondary outcome, Patient Global Impression of Change.

**Additional file 2:.** Supplementary Table S1 and Supplementary Table S2. Demographic and clinical details of the participants in the real treatment group; Demographic and clinical details of the participants in the sham treatment group.

## Data Availability

All data generated or analyzed during this study are included in this published article and its supplementary information files.

## Abbreviations

ANOVA: Analysis of variance
COG: Center of gravity
MDS-UPDRS: Movement Disorder Society Unified Parkinson’s Disease Rating Scale
MoCA: Montreal Cognitive Assessment
