# Supplementary material for: Two weeks of twice-daily prism adaptation treatment does not improve posture or gait in Parkinson’s disease: a double-blind randomized controlled trial
Source: Trials. 2021 Nov 25;22:846. doi: 10.1186/s13063-021-05832-2 (PMC8620921; doi:10.1186/s13063-021-05832-2)
Supplement: Supplementary file 2 — Additional file 2:. Supplementary Table S1 and Supplementary Table S2. Demographic and clinical details of the participants in the real treatment group; Demographic and clinical details of the participants in the sham treatment group. [file 13063_2021_5832_MOESM2_ESM.pdf]

Supplementary Table 1. Demographic and clinical details of the participants in the real treatment group. M=male; F=female; L=left; R=right; MOCA=Montreal Cognitive Assessment; MDS-UPDRS-III = Motor Disease Society Unified Parkinson's Disease Rating Scale, Part III; H & Y = Hoehn and Yahr Rating Scale; TUG = Timed up and Go; CN = Cranial Nerve; * = patient with comorbid diagnosis of Progressive Supranuclear Palsy, -= data not available / not tested.

Participant	Sex	Age (years)	Handedness	Time since diagnosis (years)	Weight (Lbs)	Height (inches)	MOCA (/30)	MDS-UPDRS-III				H & Y	TUG (s)	Medication [drug name (mg/day)]	Neurological examination
								Gait (/4)	Freezing (/4)	Postural Stability (/4)	Total (132)				
VA002	F	60	R	0.37	113	62	29	1	1	0	18	2	6.6	rasagiline (1)	Normal; CN1 not tested.
VA004	M	76	R	14.97	171	70	22	1	1	1	43	2	8.5	trihexyphenidyl (8), carbidopa (18.8), levodopa (75), pramipexole (3); selegiline (10)	Mild right shoulder weakness (1), hypoactive muscle stretch reflexes bilaterally
VA010	F	79	R	2.17	96	62	20	1	0	2	34	3	27.0	carbidopa (125), levodopa (500), pramipexole (0.25)	Mild sensory loss for pin prick sensation in bilateral lower limbs
VA012	M	73	R	4.31	345	75	17	1	0	1	24	1	13.7	carbidopa (150), levodopa (600), pramipexole (3), entacapone (600)	Hyperactive muscle stretch reflexes in arms bilaterally
VA013	M	68	R	10.32	160	65	28	2	2	1	54	3	6.8	entacapone (600), amantadine (200), carbidopa (75), levodopa (300)	Normal
VA015	M	62	R	18.96	221	68	27	1	0	1	34	2.5	8.9	carbidopa (175), levodopa (1450), ropinirole (5), selegiline (10), entacapone (1000)	CN1 abnormal (could not detect coffee ground)
VA017	F	67	R	1.26	144	65	24	1	0	1	32	2	13.3	carbidopa (100); levodopa (400); clonazepam (.5?)	No notes available
VA020	M	78	R	4.67	216	71	26	2	1	1	31	2	12.60	carbidopa (50), levodopa (200); rasagiline (1)	Impaired sense of smell
VA021	M	84	R	3.13	200	67	20	1	0	1	37	2	16.2	carbidopa (25), levodopa (100)	UPSIT 11/40; mild apgaze palsy; hypoactive R &

															L arms & legs
VA022	F	63	R	8.36	119	63	-	2	0	2	30	2	14.0	carbidopa (50), levodopa (200) 1 qhs and 1/2 qam; carbidopa (25); levodopa (80)	Poor sense of smell, mild reduction in vibration sense in R & L legs
VA024	M	58	R	10.88	134	66	29	1	0	3	26	3	8.0	carbidopa levadopa (10-100 qid, 5-200 CR qid);	Normal
VA025	F	73	R	9.38	155	59	25	2	1	3	37	3	11.32	carbidopa levadopa (100-400), ER (2) at bedtime	CN I abnormal
VA028	M	78	R	18.07	178.5	62	25	1	0	1	35	2	8.0	carbidopa/levodopa (25/100 x 2, 5x/day), azilect (), entacapone (1000), carbidopa/levadopa (50-200 qhs)	CN I abnormal, decreased vibration sense in R and L leg, hypoactive reflexes R and L arm, R and L leg)
VA029	M	69	R	3.21	155	71	25	2	1	1	55	3	11.4	rasagine (0.5), exelon (3), miralax (1700), Vit D (4), Glucosamine/chondroitin (1tab), Vit C (500),	Normal, CN 1 not tested
VA030	M	65	R	4.96	140	67	29	1	0	1	33	3	10.8	sinemet (25/100, 3x/day), bupropion (150), dulare (10), Vit D (5,000)	Mild ophthalmoparesis in all directions of gaze, could not bury the sclera on horizontal gaze
VA031	M	80	R	4.33	188	72	24	1	0	0	25	2.5	9.2	amantadine (100); carbidopa (25); levodopa (100)	Cannot smell coffee, slight cerebellar tumor (tremor?), decreased vibration in ankles (R and L)
Mean (SD)		70.8 (7.9)		7.5 (5.8)	171.0 (58.5)	66.6 (4.4)	24.7 (0.5)	1.4 (0.5)	0.4 (0.6)	1.3 (0.9)	34.3 (9.9)	2.4 (0.6)	11.6 (5.0)		

Supplementary Table 2. Demographic and clinical details of the participants in the sham treatment group. M=male; F=female; L=left; R=right; MOCA=Montreal Cognitive Assessment; MDS-UPDRS-III = Motor Disease Society Unified Parkinson's Disease Rating Scale, Part III; H & Y = Hoehn and Yahr Rating Scale; TUG = Timed up and Go; CN = Cranial Nerve; -= data not available / not tested.

Ppt	Sex	Age (years)	H'ness	Time since diagnosis (years)	Weight (Lbs)	Height (inches)	MOCA (/30)	MDS-UPDRS-III				H & Y	TUG (s)	Medication [drug name (mg/day)]	Neurological examination
								Gait (/4)	Freezing (/4)	Postural Stability (/4)	Total (132)				
VA001	F	65	R	6.25	150	63	24	0	0	0	36	2	8.4	carbidopa (150), levodopa (600), ropinirole (12); rasagiline (1)	Normal
VA005	F	60	R	6.13	186	65	27	1	0	1	23	2.5	8.2	pramipexole (2.25), carbidopa (75), levodopa (300)	Normal
VA006	M	78	R	12.81	197	67	19	2	1	3	59	3	15.4	carbidopa (200), levodopa (800)	Normal
VA007	F	63	R	21.78	120	63	26	1	0	3	45	3	11.6	carbidopa (19); levodopa(75); rasagiline (0.5); trihexiphenydil (4)	Decreased sensation to pain and vibration in left arm, Muscle stretch reflect absent in right bicep
VA008	F	70	R	6.16	116	58	28	1	0	1	41	2.5	9.0	carbidopa (125), levodopa (500), rotigotine (8), rasagiline (1)	Action tremor in hands bilaterally
VA011	M	72	L	2.5	179	74	28	1	0	1	53	3	9.2	carbidopa (850), levodopa (1150)	CN II IV VI abnormal (mild lateral gaze paresis), CN VIII abnormal (decreased hearing to finger rubs on L), decreased vibration sense on R LE
VA014	M	68	R	9.08	127	65	27	1	1	1	28	3	7.4	Carbidopa (??); levodopa (???); ropinerole (2); clonazepamm (0.5);	CN VII abnormal (R lower face does not elevate)

														gabapentin (300)	
VA016	M	72	L	7.44	213	72	30	1	0	1	19	2	6.9	carbidopa (200); levodopa (800); ropinirole (6); selegiline (10)	CN 1 abnormal (microsmia diagnosed 2011)
VA018	F	67	R	6.19	138	68	26	1	0	0	19	2	9.0	carbidopa (100); levodopa (400); trihexyphenydyil (3)	Normal
VA023	F	74	R	3.68	124	62	29	2	0	1	41	3	11.9	carbidopa (100); levodopa (400); selegiline (10); pramipexole (1.125)	Poor sense of smell (CNI); mild reduced vibration sense lower limbs bilaterally, hypoactive muscle stretch reflexes upper and lower limbs bilaterally
VA026	M	52	R	0.79	230	75	24	1	0	0	34	2	9.1	pramipexole (0.75); rasagiline (1)	mild terminal tremor (intention), heel-to-shin R leg abnormal)
VA027	F	63	L	10.54	131	63	30	0	0	0	6	2	6.4	Carbidopa (250); levodopa (1000); melatonin (5); aspirin (81); fish oil (1); estradiol (0.025); rasagiline (1), rotigotine (6), entacapone (600)	No notes available
VA032	M	84	R	7.36	168	70	26	2	1	1	31	3	15.7	Carbidopa (25; levodopa (100)	CN I abnormal, decreased temp/vibration sensitivity in legs (R and L)
Mean (SD)		68.3 (8.2)		7.7 (5.3)	159.9 (38.2)	66.5 (5.1)	26.5 (3.0)	1.1 (0.6)	0.2 (0.4)	1.0 (1.0)	33.5 (14.7)	2.5 (0.5)	9.9 (3.0)		
